# Quality of Life Is Influenced by Body Weight, Education, and Family Income in Adolescents with Chronic Diseases

**DOI:** 10.1155/2018/8485103

**Published:** 2018-10-24

**Authors:** João Felipe Mota, Marília Arantes Rezio, Ronyson Camilo Soares, Gustavo Duarte Pimentel, Alexandre Siqueira Guedes Coelho, Juliana Cunha

**Affiliations:** ^1^Clinical and Sports Nutrition Research Laboratory (Labince), Faculty of Nutrition, Federal University of Goiás, Goiânia, GO, 74.605-080, Brazil; ^2^Plant Genetics and Genomics Laboratory, Agronomy College, Federal University of Goiás, Goiânia, GO, 74690-900, Brazil

## Abstract

**Objective:**

To evaluate the health-related quality of life (HRQoL) of adolescents diagnosed with different chronic conditions and to identify demographic, socioeconomic, and health-status outcomes associated with the impairment in HRQoL.

**Study Design:**

Cross-sectional study.

**Methods:**

We evaluated 276 adolescents (50.7% male) aged 14 ± 2 years that were assisted by healthcare public service and diagnosed with cancer (CA), type 1 diabetes mellitus (DM1), overweight (OW), asthma (AS), and no chronic health condition—control group (CG). Adolescents and parent-proxy completed age-appropriate self-report and/or parent-proxy report on generic HRQoL measures using* PedsQL*™.

**Results:**

Adolescents with CA had lower overall HRQoL as well as poorer scores in all dimensions than either healthy participants or other chronic disease sufferers. HRQoL scores reported by parent-proxy were similar to those reported by adolescents across all chronic diseases. CG members reported better scores in all dimensions. Maternal education, family income, and marital status of parents were correlated with HRQoL scores in all dimensions. The risk of having an affected HRQoL score was higher in adolescents with CA than in adolescents with other chronic diseases.

**Conclusions:**

The likelihood of cancer affecting HRQoL was higher when compared to other chronic diseases, and the OW group had a worse overall score compared to CG. Adolescents with CA, AS, and OW reported worse school dimensions when compared to healthy adolescents. The education of adolescents and their parent-proxy, body weight, and family income influence the dimensions of HRQoL in adolescents with chronic diseases.

## 1. Introduction

Chronic diseases during in adolescence may cause physical, psychological, social, and school functioning damage, as well as dependency on constant therapies and hospitalizations. These repercussions have negative impacts on learning, cognitive ability, and psychosocial relationships [1, 2], affecting the family universe, leading to family conflict and impaired health-related quality of life (HRQoL) in patients and family member [3, 4].

The evaluation of HRQoL enables verification of the impact of chronic diseases in the life of the patients and their relatives and supporting professionals in the planning of treatments individualized by chronic disease. The results of this evaluation allow us to create strategies to improve adherence to treatment, reduce complications and hospitalizations, and support the construction of public policies, aiming at the improvement of HRQoL [1, 2, 5].

Factors affecting HRQoL in adolescents may differ between chronic diseases depending on the specific demands that each disease imposes on patients and their families [1, 2, 6]. Thus, the objectives of this study were to evaluate the HRQoL of adolescents diagnosed with cancer (CA), asthma (AS), type 1 diabetes mellitus (DM1), and overweight (OW) at the same time; to compare their scores with adolescents without diagnosis of diseases; to examine the convergence of the scores reported by adolescents and their caregivers; to verify the effect of time of diagnosis on HRQoL dimensions; and to identify demographic, socioeconomic, and health-status variables associated with HRQoL dimensions.

## 2. Methods

### 2.1. Study Design and Participants

We conducted a cross-sectional study with adolescents of both sexes, aged 10-18 years, and their respective caregivers. Adolescents were divided into different groups according to their medical diagnosis: CA, DM1, OW, or AS. Participants who were receiving anticancer treatment (chemo or radiation therapy and recent postoperative), classified as moderate persistent grade of AS and taking medicines, and diagnosed with DM1 or OW were considered in the active stage of disease. Adolescents diagnosed with CA and AS followed in an outpatient setting and without medications were considered in inactive/remission stage of the disease. These adolescents were interviewed during their routine visit to public healthcare services, and adolescents with more than one chronic disease were excluded from the study. We also analyzed matched controls (CG), without a chronic health condition or disease, in order to make comparisons between groups. The parent-proxy of each adolescent also completed the PedsQL questionnaire about their perceptions of the HRQOL of the adolescent.

Participants with severe mobility issues (e.g., in use of wheelchair or walker) and neurological diseases were excluded from the study. This study was approved by the Ethics Committee of the Clinics Hospital of the Federal University of Goiás (reference number 542.655/2014). All participants, adolescent and parents, were informed about the study orally and in writing and gave written informed consent to participation.

### 2.2. Measures

HRQoL was analyzed by Pediatric Quality of Life Inventory (PedsQL)™ Generic Core Scales (version 4.0) using the validated Brazilian Portuguese version which was authorized by Mapi Research Trust [7]. The PedsQL 4.0 Generic Core Scales is a 23-item questionnaire that evaluates HRQoL in the subdomains of physical (eight items), emotional (five items), social (five items), and school functioning (five items). The possible responses were measured with a 5-point Likert scale from 0 = never a problem to 4 = almost always a problem. These reverse scaled scores were then transformed to a score of 0-100 to create the overall scaled score and the subcategory scores, with higher scores representing better HRQoL [5]. The instruments consist of parallel child self-report and parent proxy-report formats. We also calculated the summary scores. The Physical Health Summary Score (8 items) is the same as the Physical Functioning Subscale. To create the Psychosocial Health Summary Score (15 items), the mean is computed as the sum of the items divided by the number of items answered in the Emotional, Social, and School Functioning Subscales [5].

A study-specific questionnaire containing socioeconomic, demographic, clinical, and lifestyle-related questions was used to characterize the sample and to make associations with the results of the PedsQL 4.0. In addition, a closed question with a “yes” or “no” option verified whether the adolescent diagnosed with any of the chronic conditions thought that his health condition interfered with his school performance.

The researchers were previously trained to standardize the collection procedure, minimize possible errors, and guarantee the quality of the data collected [8]. Due to the low level of schooling of the participants, the HRQoL surveys were administered in person by the researchers, reducing incidence of errors during completion, like the study conducted by Klatchoian et al. [9]. Adolescents and parent-proxies answered questions separately. Interviewees answered how much each item was a problem during the last month [5]. The average time to answer the questionnaire was 4.68 ± 0.84 min for adolescents and 5.09 ± 0.81 for the parent-proxy. These times were in accordance with other studies [5, 9].

The height of the adolescents was measured during the selection phase to the nearest 0.5 cm with a stadiometer (Model Standard, Sanny®). Weight was measured (after voiding, with participants wearing light clothing) to the nearest 0.1 kg on a digital scale (Filizola, Brazil). Height-for-age and body mass index-for-age indexes were calculated using the Anthro software [10] and the results analyzed using growth curves [11].

### 2.3. Statistical Analyses

To determine the sample size, we used the difference between the mean HRQoL scores of groups (10.0) and the standard deviation (16.3) which were obtained in the study of Schwimmer et al. [6], and adopted the power of 80% and the significance level of 5%. The analysis was performed using the Student-t-based approach to compare means of independent samples, known as conservative in this case in which the means of the five groups are compared by the ANOVA test. The sample size was calculated at 33 individuals per group, including a 10% loss rate, totaling 165 adolescents.

The Shapiro-Wilk, Lilliefors, and Kolmogorov-Smirnov tests were performed to evaluate the normality of residues of all variables. Descriptive statistics were calculated for the whole sample and for each group. The *α*-Cronbach coefficient was evaluated to determine the reliability of the internal consistency and its result was ≥0.83 and ≤0.90 for all dimensions of HRQoL in the adolescent-responsible pair. The average HRQoL scores for each group were compared using the one-way ANOVA procedure. When appropriate, the post hoc Tukey test was performed. Average scores as reported by the adolescents were compared to those reported by their parents using paired t tests. Simple linear regression analyses were carried out for all scores on the time since diagnosis. Multiple linear regression analyses for all scores on socioeconomic, demographic, and anthropometric variables were performed using the step-wise approach adjusted by disease. P-values lower than 0.05 were considered significant. Statistical analyses were performed in R 3.4.1. [12].

## 3. Results

### 3.1. Participants

A total of 361 adolescents were invited to participate in the study, but 85 refused because they had no interest or availability. Therefore, 276 adolescents and their parents/guardians consented to enroll in the study. Characteristics of the sample interviewed are shown in Table 1. Most the questionnaires (83.0%) were answered by the mother, 54.7% (n = 151) of adolescents lived with their parents, 36.59% (n = 101) lived with their mother alone, 92.8% (n = 256) had one sibling, 88.3% (n = 226) up to three siblings. The AS group had a lower age when compared to the other groups (P < .05). CG had a higher prevalence of parents with stable union than OW (43% vs. 76%, P < .05).

In the CA group, 44.61% (n = 29) were undergoing chemotherapy, 38.46% (n = 25) were in outpatient follow-up, 10.76% (n = 7) in radiotherapy, and 6.15% (n = 4) at the postoperative stage. The most prevalent cancers were leukemia (30.8%), lymphomas (21.5%), intracranial neoplasms (10.8%), and others (36.9%). Of the adolescents diagnosed with AS, 63.3% (n = 19) were classified as moderate persistent grade. Among OW adolescents, 24.0% (n = 12) were overweight, 48.0% (n = 24) were obese and 28.0% (n = 14) severe obese. Adolescents considered in active stage of the disease were CA (61.5%), AS (63.3%), DM1 (100%) and OW (100%).

### 3.2. Adolescent Self-Report of PedsQL

CG presented higher HRQoL and higher scores for physical health, psychosocial summary and school functioning dimensions when compared to CA (p < .05). The total score and school dimensions were, respectively, 7.6% and 10.5% lower in OW vs. CG (p < .05). The school functioning dimension was also more affected in AS vs. CG (p < .05). CA reported lower HRQoL in the physical and psychosocial dimensions when compared with DM1 (p < .05) and AS (p < .05). CA presented lower emotional dimension vs. AS (p < .05), social dimension vs. DM1 (p < .05) and school dimension vs. DM1 (p < .05) and OW (p < .05) (Table 2).

### 3.3. Parent-Proxy Report of PedsQL

When the questionnaire was reported by parent-proxy, scores on all dimensions of HRQoL were higher in CG when compared to chronic disease groups (p < .05). Comparing groups with some type of chronic disease, CA showed lower HRQoL in physical, psychosocial, and school dimensions. DM1 presented higher scores than OW for total score and physical dimension and lower scores on social and school dimensions (Table 2).

### 3.4. Differences in Self-Report and Parent-Proxy Report of PedsQL

In the CG, scores in all dimensions were higher in parent-proxy reports than in the adolescents (p < .001). The parent-proxy of adolescents with cancer reported lower scores in the emotional functioning dimension (p < .05) (Table 2).

### 3.5. Effect of Diagnostic Time on Health-Related Quality of Life

The median time of diagnosis in groups with chronic diseases was 2 (0.08–17.00) years. In general, 72.7% of participants were supported by more than two health professionals, 69.9% had had no hospitalizations in the last year, and 25.0% had had between one and five hospitalizations. OW had the lowest percentage of hospitalizations (8%) and CA the highest percentage (52.3%).

We used a simple linear regression model to evaluate the effect of the time of disease diagnosis on HRQoL and its dimensions in each chronic disease group (Table 3). This model revealed that in the OW group there is a reduction, as a function of the time of disease diagnosis, in the total score (-1.26 ± 0.58. p < .05), summary psychosocial health (-1.12 ± 0.55. P < .05), and school functioning dimensions (-1.52 ± 0.60. p < .05). On the other hand, the model also showed that there is an improvement in the physical health summary score in the CA group (1.90 ± 0.65, p < .01).

### 3.6. Effect of Demographic, Socioeconomic, Lifestyle, and Anthropometric Variables on HRQoL Dimensions

We also used a multiple linear regression model to assess the influence of socioeconomic, demographic, anthropometric, and lifestyle variables on HRQoL dimensions (Table 4). The total HRQoL score was positively influenced by the following variables: having siblings (7.44 ± 3.47, p < .05) and performing physical activity (8.23 ± 2.25, p < .001). It was negatively influenced by having widowed parents (-12.27 ± 4.01, p < .01), being a citizen of another province/ state (-8.97 ± 2.89, p < .01), and having body weight (-0.20 ± 0.07, p < .01).

Income (0.02 ± 0.003, p < .001), to be studying (13.91 ± 4.06, p < .001), and to perform physical activity (11.09 ± 2.77, p < .001) all had a positive effect on the physical health summary. A negative effect was produced by having widowed parents (-16.53 ± 5.05, p < .01), number of people in the household (-5.02 ± 1.53, p < .01), and BMI (-0.81 ± 0.26, p < .01) (Table 4).

The psychosocial health summary score was positively influenced by studying (6.62 ± 3.26, p < .05), and performing physical activity (8.68 ± 2.25, p < .001), and negatively influenced by having widowed parents (-13.17 ± 3.95, p < .01), being a migrant from another state (-7.56 ± 2.83, p < .01), and BMI (-0.46 ± 0.18, p < .05). The fact of being male had a positive effect (7.02 ± 2.72, p < .05), and citizen of another state had a negative effect (-9.37 ± 4.41, p < .05) on the emotional dimension. Living with only the mother had a negative effect on the school functioning dimension (-19.69 ± 7.66, p < .05), and BMI had a negative effect on the social dimension (-0.89 ± 0.31, p < .01).

## 4. Discussion

To our knowledge, this was the first study to evaluate the HRQoL of four different chronic diseases simultaneously, comparing them to control group and establishing correlations with demographic, socioeconomic, and lifestyle variables. Our results show important differences in HRQoL among the chronic diseases. The influence of time of disease diagnosis on HRQoL and its dimensions was established in the cancer and overweight groups. When the adolescent is studying, or performing physical activity, their HRQoL is positively affected, while adolescents' HRQol is negatively affected by having widowed parents or originating from another state/ province.

The CA group had the worst scores in all HRQoL dimensions in comparison to the other groups studied, both in the adolescent self-report and in the parent-proxy report. In addition, the adolescents in this group had low school attendance and stated that the health condition impaired school performance. These findings corroborate those of other studies, demonstrating a greater impairment of HRQoL dimensions in pediatric cancer patients [13–16]. The fact that leukemia and lymphoma were the most prevalent types of cancer in this study, and that most participants were in chemotherapy, may have contributed to this group presenting a greater impairment of HRQoL dimensions when compared to the other chronic disease groups. Speechley et al. [14] evaluated the HRQoL of 800 cancer survivors aged 6–16 and found that patients with leukemia and lymphoma had greater physical and psychosocial health impairments when compared to other types of cancer. On the other hand, some studies also show that adolescents with cancer who are in chemotherapy have worse HRQoL scores in comparison to healthy adolescents and those with other chronic conditions [15, 16]. Chemotherapy is an aggressive treatment that affects both the biological function of the patient and the patient's psychological and social health [13]. Due to immunosuppressive therapy and constant hospitalization, patients are forced to withdraw from school and have difficulty maintaining normal activities, and, consequently, impaired social interaction [6, 13, 17]. The fact that parent-proxy reports for the CA group also reported lower HRQol scores possibly arises from poor prognosis and/or aggressive therapies that affect the emotional wellbeing of the whole family [18–20].

The DM1, OW and AS groups did not present differences in HRQoL scores when compared to each other. On the other hand, when compared to the CG, the DM1, OW, and AS groups had worse scores in the school dimension, and the OW group had a worse overall score. In fact, asthma symptoms and smoke have been associated with impaired physical functioning and mental illness, and, consequently, with worse HRQoL scores [21]. In outpatient follow-ups, adolescents with asthma and type 1 diabetes have better clinical control of the disease, and serious complications may only arise years after diagnosis [22, 23].

In our study, mean time to diagnosis of diabetes was 6.5 ± 3.8 and asthma 7.0 ± 4.0 years, which is not considered recent. In addition, patients receiving adequate treatment can have a good prognosis, use different strategies to normalize the routine, minimize the impact of the disease and, consequently, present better HRQoL [23, 24].

A study of 2,101 adolescents aged 10–18 from 17 countries in Europe, Japan, and North America found that metabolic control of DM1 in adequately monitored patients is associated with improved quality of life [25].

Obesity in adolescence is associated with lower scores of HRQoL [26, 27]. This is a stigmatizing condition, poorly accepted socially [6, 28], and is often not considered a disease by patients and their parent-proxy, which results in a lack of and low adherence to treatment. Interestingly, in our study, adolescents in the OW group and their parent-proxy reported that they were being followed up on an outpatient basis because of the quality of their food and because they had a disease. However, when followed up, obese adolescents are usually submitted to intensive medical interventions and experience numerous adverse effects of drug treatments, which may influence their quality of life [6].

In the present study, the multiple linear regression showed that the performance of physical activity has a positive effect on total score, physical health, and psychosocial health. However, Rank et al. [29] found that the positive effect of physical activity on HRQoL scores may be present in overweight adolescents who take part in follow-up weight-loss programs, whether or not these result in changes in body composition, such as BMI.

In the multilinear regression model of our study, we found that a higher BMI value is associated with a lower score in the physical health and psychosocial health summaries, as well as in the social dimension. Obese patients suffer constant verbal abuse due to their physical condition and have significantly affected social dimensions [28, 30, 31]. In a previous study, Han et al. [32] found a correlation between the psychosocial, social, and physical dimensions of obese youths and low self-esteem.

Another relevant finding was that adolescents with chronic diseases had better physical health and social functioning scores if they were from families with higher incomes. The family income of a patient with a chronic disease is compromised by factors related to treatment such as transportation, medication acquisition, and meals outside the home on days of consultations and hospitalizations [33]. Families with higher incomes feel these impacts less [34, 35], which could correlate with better scores in these dimensions.

The educational level of the parent-proxy was positively correlated with scores on HRQoL dimensions of adolescents with chronic diseases, with an improvement in the school dimension in CA and AS groups. The low level of schooling of parents or guardians may compromise treatment, since the complexity of the therapy requires cognitive skills that are often not present [34]. People with higher levels of education have greater knowledge and cognitive ability, which may allow a better understanding of the health condition and increase the probability of adherence to treatment [35].

The scores in the emotional and social dimensions did not differ between CG and groups with chronic disease, whereas the psychosocial health summary score differed only in the CA group. On the other hand, while patients with a chronic disease may have dimensions of HRQoL affected by their health condition, adolescents without diseases may also present difficulties in the different dimensions due to factors relating to societal changes. In our study, adolescents in the control group reported worse scores in all dimensions when compared to the parent-proxy report.

The main limitations of this study in our perception were the inclusion of different diseases and the absence of parameters to detail the disease status. Longitudinal and routine studies in the health services are necessary to better clarify the cause-and-effect relationships and the ideal moment for intervention. In addition, some groups did not have many participants, despite efforts to maximize recruitment.

In conclusion, the likelihood of cancer affecting HRQoL was higher when compared to other chronic diseases, and the OW group had a worse overall score compared to CG. Adolescents with cancer and asthma or who were overweight reported worse school dimensions when compared to healthy adolescents. The education of adolescents and their parent-proxy, body weight, and family income influence the dimensions of HRQoL.

These results can help health professionals, school institutions, and legislators to attend more carefully to the biopsychosocial issues of adolescents with chronic diseases and to plan early interventions accordingly. In addition, we suggest the need for specific strategies and support networks that focus on the biopsychosocial issues of patients with chronic diseases and their families.

## Figures and Tables

**Table 1:** Baseline characteristics of the sample per group of chronic diseases.

**Variables**	**Groups**					
	**CG**	**CA**	**DM1**	**OW**	**AS**	**All groups**
**N**	100	65	31	50	30	276
**Diagnostic time **(M ± SD)	-	3.01 ± 3.8	6.4 ± 3.8	2.6 ± 3.1	7.0 ± 4.0	4.1 ± 4.1
**Age **(M ± SD)	14.1 ± 1.8	13.56 ± 2.5	14.6 ± 2.6	13.3 ± 2.0	11.5 ± 2.1^*∗*^	13.6 ± 2.3
**Sex **(n, %)						
Female	57 (57.0)	28 (43.1)	17 (54.8)	25 (50.0)	09 (30.0)	136 (49.3)
Male	43 (43.0)	37 (56.9)	14 (45.2)	25 (50.0)	21 (70.0)	140 (50.7)
**Marital status **(% N)						
Stable union	43 (43.0)^b^	34 (52.3)	22 (71.0)	38 (76.0)	19 (63.3)	156 (56.5)
Divorced	49 (49.0)	26 (40.0)	6 (19.4)	11 (22.0)	11 (36.7)	103 (37.3)
Widower	8 (8.0)	5 (7.7)	3 (9.6)	1 (2.0)	0 (0.0)	17 (6.2)
**Nutritional diagnosis **(% N)						
Malnutrition	2 (2.0)	8 (12.3)	2 (6.5)	-	0 (0.0)	12 (4.3)
Eutrophy	84 (84.0)	43 (66.2)	25 (80.6)	-	23 (76.7)	175 (63.4)
Overweight	14 (14.0)	14 (21.5)	4 (12.9)	50 (100)^*∗*^	7 (23.3)	89 (32.2)

M = mean; SD = standard deviation; CA = cancer group; DM1 = type 1 diabetes group; OW = overweight group; CG = control group; ^a^*p* < .001 vs all groups based on Tukey *post hoc* ANOVA.

**Table 2:** Comparison of total scores and HRQoL dimensions by chronic disease groups and control group, adolescent self-report and parent-proxy report.

**Scale**	**Groups**					**Differences from CG** ^**∗**^	**Differences between chronic disease groups** ^**∗**^
	**CG**	**CA**	**DM1**	**OW**	**AS**		
**Self-Report (Adolescent)**	N = 100	N = 65	N = 31	N = 50	N = 30		
Total score	77.70 (10.45)^†††^	67.58 (13.03)^†††^	76.85 (11.92)^†^	71.8 (13.34)^†††^	76.67 (13.90)^†^	CG> CA, OW	CA < DM1, AS
Physical health	82.56 (10.78)^†††^	68.17 (20.94)^†††^	81.75 (14.62)^†††^	75.62 (15.31)^†^	79.17(14.34)^†^	CG> CA	CA < DM1, AS
Psychosocial health	74.80 (10.43) ^†††^	63.67 (13.16)^†††^	72.47 (12.36)	69.21 (12.71)^†††^	73.29 (14.08)^†^	CG> CA	CA < DM1, AS
Emotional functioning	65.00 (15.94) ^†††^	62.62 (17.70)	64.52 (18.59)	63.20 (18.92)^†^	73.67 (17.51)^†^	-	-
Social functioning	86.40 (14.82) ^†††^	79.31 (18.50)^†^	90.00 (16.88)	79.50 (20.13)^†††^	86.33 (17.24)	-	CA < DM1
School functioning	76.85 (12.65) ^†††^	59.92 (18.80)^†††^	71.12 (17.73)^††^	68.80 (14.09)^†††^	67.17 (17.40)	CG> CA, OW, AS	CA < DM1, OW
**Proxy-Report**							
Total score	85.33 (10.15)	65.30 (15.55)	76.90 (11.54)	68.08 (16.57)	73.62 (12.33)	CG> all	CA < DM1, AS/ DM1 >OW
Physical health	89.65 (09.58)	64.04 (24.82)	82.26 (17.55)	69.50 (22.50)	77.81 (17.91)	CG> CA, OW, AS	CA < DM1, AS/ DM1 >OW
Psychosocial health	83.88 (11.26)	65.59 (14.69)	75.11 (11.82)	67.60 (16.29)	72.22 (13.48)	CG> all	CA < DM1
Emotional functioning	76.10 (16.20)	55.69 (19.50)	59.68 (18.53)	62.30 (18.38)	66.00 (21.55)	CG> CA, DM1, OW	-
Social functioning	93.35 (12.31)	79.08 (22.46)	89.03 (15.99)	75.80 (24.98)	85.00 (17.62)	CG> CA, OW	DM1 <OW
School functioning	82.20 (14.55)	62.00 (21.37)	76.61 (14.51)	64.70 (22.21)	65.67 (17.21)	CG> CA, OW, AS	CA < DM1 / DM1 <OW

Data were expressed in mean and standard deviation.

CG = control group; CA = cancer group; DM1 = type 1 diabetes group; OW = overweight group.

^**∗**^ *p* < .05 based on Tukey *post hoc *ANOVA; ^†^*p* < .05; ^††^*p<* 0.01; ^†††^*p<* 0.001 based on pared t-test.

**Table 3:** Influence of the time since diagnosis on the HRQoL dimensions by group of chronic diseases.

**Groups**	**HRQoL Dimensions**					
	**Total Score**	**Physical health**	**Psychosocial health**	**Emotional functioning**	**Social functioning**	**School functioning**
Cancer	0.50 ± 0.42	1.90 ± 0.65^*∗∗*^	0.52 ± 0.43	-0.16 ± 0.58	0.41 ± 0.61	-0.12 ± 0.62
Type 1 diabetes mellitus	-0.12 ± 0.58	-0.19 ± 0.71	-0.40 ± 0.60	0.10 ± 0.91	0.72 ± 0.81	-1.09 ± 0.84
Overweight	-1.26 ± 0.58^*∗*^	-0.76 ± 0.69	-1.12 ± 0.55^*∗*^	-1.10 ± 0.84	-1.67 ± 0.88	-1.52 ± 0.60^*∗*^
Asthma	0.75 ± 0.64	0.32 ± 0.67	0.71 ± 0.65	0.38 ± 0.82	0.88 ± 0.79	1.50 ± 0.77

Data were expressed in mean and standard deviation.

^*∗*^ *p* < .05; ^*∗∗*^*p* < .01 based on simple linear regression.

**Table 4:** Effect of socioeconomic, demographic, and anthropometric variables on HRQoL dimensions of the total sample (n = 276).

	**Variables**	**HRQoL dimensions**					
		**Total Score**	**Physical health**	**Psychosocial health**	**Emotional functioning**	**Social functioning**	**School functioning**
**Socioeconomic**	**Parent-proxy education ESC**	-	-	-	-	39.22 ± 12.80^*∗∗*^	-
	**Parent-proxy education HSC**	-	-	-	-	-	-
	**Parent-proxy education IHE**	26.50 ± 9.97^*∗∗*^	-	32.25 ± 9.89^*∗∗*^	-	-	56.42 ± 18.78^*∗∗*^
	**Parent-proxy education FHE**	-	-	-	-	-	38.82 ± 17.76^*∗*^
	**Widowed parents**	-12.27 ± 4.01^*∗∗*^	-16.53 ± 5.05^*∗∗*^	-13.17 ± 3.95^*∗∗*^	-	-	-
	**Separated parents**	-	-	-	-	8.92 ± 3.10^*∗∗*^	-
	**Live with mother only**	-	-	-	-	-	-19.69 ± 7.66^*∗*^
	**Income**	-	0.02 ± 0.003^*∗∗∗*^	-	-	0.008 ± 0.002^*∗∗*^	-
	**Studying**	-	13.91 ± 4.06^*∗∗∗*^	6.62 ± 3.26^*∗*^	-	-	-

**Demographic**	**Citizen of another province/ state**	-8.97 ± 2.89^*∗∗*^	-	-7.56 ± 2.83^*∗∗*^	-9.37 ± 4.41^*∗*^	-14.59 ± 4.02^*∗∗∗*^	-8.80 ± 4.07^*∗*^
	**Male**	-	-	-	7.02 ± 2.72^*∗*^	-	-
	**Have brothers**	7.44 ± 3.47^*∗*^	-	-	-	11.19 ± 5.18^*∗*^	-
	**Number of people in the household**	-	-5.02 ± 1.53^*∗∗*^	-	-	-	-

**Anthropometric**	**Perform physical activity**	8.23 ± 2.25^*∗∗∗*^	11.09 ± 2.77^*∗∗∗*^	8.68 ± 2.25^*∗∗∗*^	-	-	-
	**Weight**	-0.20 ± 0.07^*∗∗*^	-	-	-	-	-
	**BMI**	-	-0.81 ± 0.26^*∗∗*^	-0.46 ± 0.18^*∗*^	-	-0.89 ± 0.31^*∗∗*^	-

Data were expressed in mean and standard deviation. ESC: Elementary School Complete; HSC: High School Complete; IHE: Incomplete Higher Education; FHE: Full Higher Education. ^*∗*^*p* < .05; ^*∗∗*^*p* < .01; ^*∗∗∗*^*p* < .001 based on multiple linear regression.

## Data Availability

The data used to support the findings of this study are available from the corresponding author upon request.
